# Decoding the microbiome-immune crosstalk in cancer: from mechanisms to therapeutic translation

**DOI:** 10.1186/s40364-026-00930-2

**Published:** 2026-05-27

**Authors:** Ning Zhao, Liang Wu, Sha Peng, Hui Yang, Yongchun Song, Yong Zhang, Lingwen Ding

**Affiliations:** 1 Department of Surgical Oncology, First Affiliated Hospital of Xi’an JiaoTong University, Xi’an, 710061 China; 2 Department of Pathology, Yong Loo Lin School of Medicine, National University of Singapore, Singapore, Singapore; 3 Department of Gastric Surgery, Division of Life Sciences and Medicine, The First Affiliated Hospital of USTC, University of Science and Technology of China, Hefei, 230001 China; 4 Department of Oncology Rehabilitation, Shaanxi Provincial Rehabilitation Hospital, Xi’an, China

**Keywords:** Gut microbiota, Immunotherapy resistance, Microbiota-targeted intervention, Tumor immune microenvironment

## Abstract

The gut microbiome plays a critical role in shaping host immunity and profoundly affects the efficacy of cancer immunotherapy. Accumulating evidence suggests that interventions designed to alter the microbial community, including fecal microbiota transplantation, probiotics, and engineered bacteria, can reprogram the tumor-immune microenvironment and enhance clinical efficacy. This Review provides a comprehensive overview of the molecular and cellular mechanisms through which the gut microbiota influences antitumor immunity, and it highlights recent clinical studies evaluating these interventions. We further examine inherent challenges, including inter-individual variability in microbial composition, difficulties in achieving stable and durable colonization, technical barriers in delivery, and potential safety concerns associated with immune activation or off-target effects. Finally, we discuss future directions for translating microbiome-targeted therapies into oncology, emphasizing the need for mechanistic insight, standardized protocols, rigorous evaluation, and integration with precision immunotherapy strategies to optimize therapeutic outcomes.

## Clinical trial number

Not applicable.

## Introduction

 The advent of cancer immunotherapy, particularly immune checkpoint inhibitors (ICIs), has fundamentally transformed the therapeutic landscape of oncology by harnessing the host’s immune system to recognize and eliminate malignant cells. Agents targeting cytotoxic T-lymphocyte-associated protein 4 (CTLA-4) and the programmed cell death (PCD) protein 1/ligand 1 (PD-1/PD-L1) axis have yielded unprecedented responses in subsets of patients across various advanced cancers [1–3]. However, durable responses remain limited, with most solid tumors exhibiting overall response rates of approximately 20–30% [4, 5]. In addition, generalized immune activation can result in immune-related adverse events (irAEs), ranging from manageable dermatologic or gastrointestinal toxicities to life-threatening conditions such as myocarditis and pneumonitis [6–9]. These limitations underscore the urgent need to identify determinants of ICIs response and to develop strategies that enhance efficacy while minimizing toxicity.

Traditionally, efforts to define predictive factors for ICIs response have focused on tumor-intrinsic and microenvironmental features [10]. Oncogenic driver mutations [11], tumor mutational burden [12], and the density and phenotype of tumor-infiltrating lymphocytes (TILs) [13] have been extensively studied as biomarkers. While insightful, this tumor-centric perspective cannot fully explain the heterogeneity in patient outcomes, prompting broader investigations into systemic host factors that may influence anti-tumor immunity. Among these, gut microbiota has been highlighted as a critical contributor within a distinct ecological niche [14, 15]. The human gastrointestinal tract contains a diverse, complex, and dynamic community of bacteria, viruses, and fungi, collectively known as the gut microbiota. Acting as a virtual endocrine organ, the microbiota shapes immune homeostasis from early life, providing essential signals for the development and education of both innate and adaptive immune cells [16–20], thereby establishing systemic immune vigilance and tolerance.

The causal link between the gut microbiota and ICIs efficacy was first demonstrated in murine models, where modulation of the gut microbiome profoundly influenced tumor growth and responsiveness to anti-PD-1 therapy [21, 22]. Clinical observations corroborated these findings, showing that patients receiving broad-spectrum antibiotics shortly before or during ICIs treatment exhibited significantly worse overall (OS) and progression-free survival (PFS) across multiple cancer types [23–26]. High-resolution metagenomic analyses further identified distinct microbial signatures distinguishing responders from non-responders, with beneficial taxa such as Faecalibacterium prausnitzii [27], Bifidobacterium longum [28], Akkermansia muciniphila [29], and members of the Ruminococcaceae and Lachnospiraceae families [30] consistently enriched in responders. Importantly, fecal microbiota transplantation (FMT) from responding patients or healthy donors into non-responding patients conferred enhanced tumor control and responsiveness to ICIs [31, 32], providing direct evidence for microbiota-mediated modulation of antitumor immunity.

These findings have established the foundation of oncomicrobiomics, recognizing that ICIs outcomes are shaped not only by the tumor microenvironment (TME) but also by systemic crosstalk with the gut microbiota. Current research reveals that this interplay operates through multiple axes, including: (i) bacterial components, such as lipopolysaccharides (LPS) [33] and flagellin [34], engaging pattern recognition receptors (PRRs) on immune cells to provide basal stimulation that enhances responsiveness to ICIs; (ii) antigenic mimicry, whereby T cells primed against microbial epitopes cross-react with homologous neoantigens expressed by tumor cells, creating a pre-existing pool of effector cells poised for action upon immune checkpoint blockade (ICB) [35]; and (iii) microbial metabolites, such as short-chain fatty acids (SCFAs), secondary bile acids, and tryptophan catabolites, can reach the systemic circulation and affect immune cell function via specific receptors or epigenetic mechanisms, thereby shaping effector activity within the TME [36, 37].

The rapid accumulation of mechanistic insights has driven the development of microbiota-targeted interventions aimed at enhancing ICIs efficacy. These approaches range from dietary modulation to administered live biotherapeutics, including defined probiotic consortia [38] and engineered microbial strains [39], as well as more profound ecological manipulations such as FMT [40]. Each strategy presents unique opportunities and challenges, and many are advancing from preclinical validation to early-phase clinical trials, heralding a new era of microbiome-enhanced oncology.

This review aims to provide a comprehensive overview of the field, focusing on the mechanisms by which the gut microbiota modulates response to cancer immunotherapy. We also evaluate the current microbiota-targeted interventions, considering both their therapeutic potential and limitations. Finally, we discuss major challenges hindering clinical translation, including standardization, personalization, and trial design, and provide perspectives on leveraging the microbiome to improve cancer outcomes.

## Underlying mechanisms of gut microbiota in modulating cancer immunotherapy

**Fig. 1:**
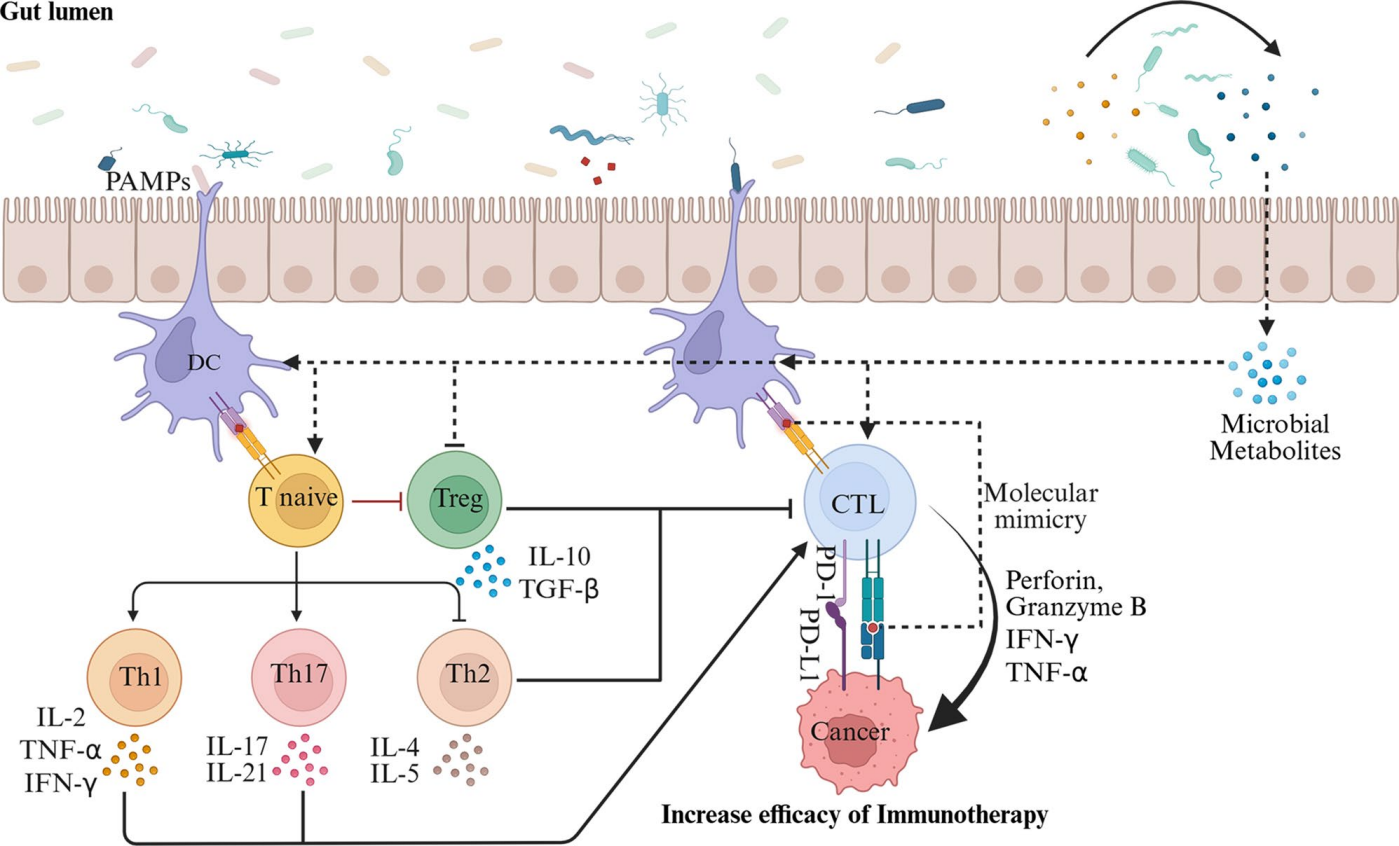
Mechanisms by which targeting the gut microbiota enhances anti-tumor immune responses. The gut microbiota can promote CTL-mediated tumor cell killing through multiple mechanisms, including the recognition of PAMPs by PRRs, molecular mimicry, and microbiota-derived metabolites. CTL: cytotoxic T lymphocyte; PAMPs: pathogen-associated molecular patterns; PRRs: pattern recognition receptors; IL: interleukin; TGF-β: transforming growth factor-β; TNF-α: tumor necrosis factor-α; IFN-γ: interferon-γ; PD-1: programmed cell death protein 1. Original figure created with https://BioRender.com

The strong correlation between gut microbial composition and response to ICIs has shifted from observation to a central paradigm in oncology. Moving beyond correlation, it is crucial to delineate the molecular and cellular pathways through which commensal bacteria modulate systemic anti-tumor immunity. This section dissects these mechanisms, illustrating how the gut microbiota functions as a master regulator of immune responses (Fig. 1).

### Immune system priming and functional modulation by gut microbiota

The gut microbiota continuously educates and primes host immune cells throughout life, establishing a basal immune tone that is important for effective immune responses and long-term immune homeostasis [18, 20, 41]. Early-life colonization by commensal bacteria plays a crucial role in shaping the development of the host immune system, influencing the maturation of dendritic cells (DCs), macrophages, neutrophils, and lymphocyte subsets [19, 42]. This lifelong priming largely depends on microbial-associated molecular patterns (MAMPs) recognized by PRRs. Key bacterial components include LPS from Gram-negative bacteria, lipoteichoic acid (LTA) and lipopeptides from Gram-positive bacteria, peptidoglycan fragments, flagellin, and bacterial nucleic acids such as unmethylated CpG DNA or double-stranded RNA.

Membrane-bound PRRs mediate recognition of extracellular ligands: TLR4, in cooperation with CD14 and MD-2, senses LPS [43]; TLR2, often forming heterodimers with TLR1 or TLR6, detects LTA and di- or tri-acylated lipopeptides [44]; TLR5 recognizes extracellular flagellin [45]; and endosomal TLR9 senses bacterial unmethylated CpG DNA [46], while TLR3 and TLR7/8 detect bacterial double- [47] or single-stranded RNA [48] delivered to endosomes. Ligand binding activates MyD88- or TRIF-dependent NF-κB, MAPK, and IRF pathways, driving the transcription of pro-inflammatory cytokines such as TNF-α, IL-6, IL-1β, and IFN-γ, which in turn promote DCs maturation, cytotoxic T lymphocyte (CTL) recruitment and activation, and restrain regulatory T cell (Tregs) activity, shifting the immune microenvironment toward an immunostimulatory state [49, 50].

Cytosolic sensors complement this system: peptidoglycan fragments and muramyl dipeptides are recognized by NOD1 and NOD2, triggering RIP2-TAK1-NF-κB/MAPK signaling, inducing type I interferons and chemokines, and linking innate microbial sensing to adaptive T cell priming [51, 52]. Intracellular flagellin or certain peptidoglycan species activate NAIP/NLRC4 and NLRP3 inflammasomes, resulting in Caspase-1-mediated pyroptosis and IL-1β/IL-18 release [53]. Bacterial nucleic acids that reach the cytoplasm are sensed by cGAS, RIG-I, MDA5, and AIM2/IFI16, leading to STING- or MAVS-dependent IRF3/7 activation, type I interferon production, and inflammasome-mediated pro-inflammatory responses [54, 55].

**Fig. 2:**
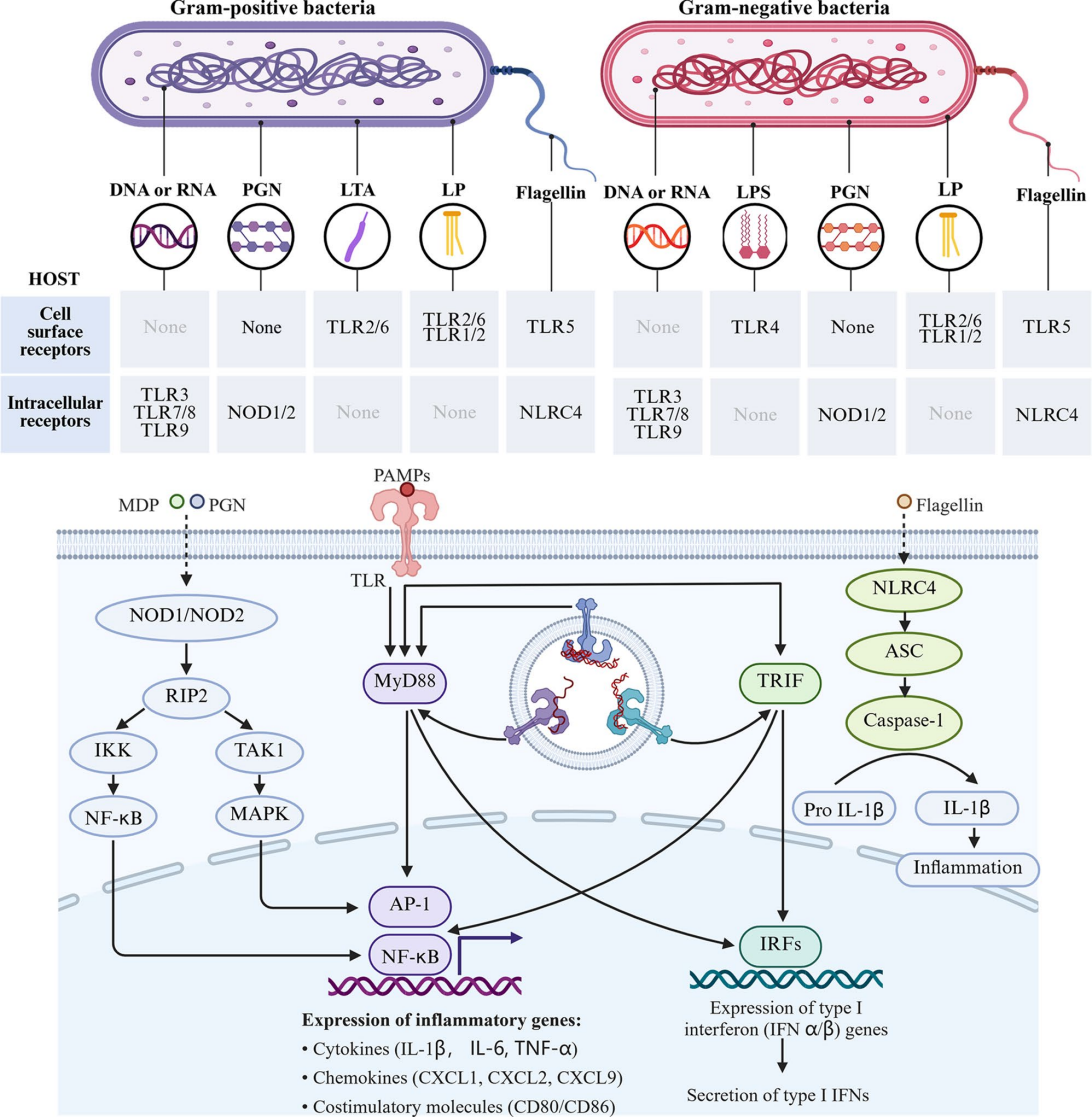
The microbiota provides diverse PAMPs that are detected by pattern recognition receptors on or inside host immune cells. Once recognized, multiple signaling pathways are activated, leading to NF-κB and IRF activation, cytokine and interferon production, and subsequent immune cell recruitment, activation, maturation, and antigen presentation to trigger immune response. PAMPs: pathogen-associated molecular patterns; PGN: peptidoglycan; LTA: lipoteichoic acid; LP: lipoprotein; LPS: lipopolysaccharide; TLR: toll-like receptor; MDP: muramyl dipeptide; NOD: nucleotide-binding oligomerization domain-containing protein; ASC: apoptosis-associated speck-like protein containing a CARD; RIP2: receptor-interacting protein kinase 2; IKK: IκB kinase; TAK1: transforming growth factor-β–activated kinase 1; MAPK: mitogen-activated protein kinase; AP-1: activator protein 1; MyD88: myeloid differentiation primary response 88; TRIF: TIR-domain-containing adapter-inducing interferon-β; NLRC4: NLR family card domain containing 4; IRF: interferon regulatory factor; TNF-α: tumor necrosis factor-α; IL: interleukin; CXCL: C-X-C motif chemokine ligand; CCL: C-C motif chemokine ligand; IFN: interferon; CD: cluster of differentiation. Original figure created with https://BioRender.com

Through this multi-layered and continuous low-grade stimulation throughout life, the gut microbiota shapes immune development, maintains innate immune readiness, enhances antigen presentation, promotes effector T cell function, and skews myeloid cells toward a pro-inflammatory phenotype (Fig. 2). Collectively, these interactions position the gut microbiota as an endogenous adjuvant, calibrating the magnitude and quality of host anti-tumor immunity and underpinning the lifelong establishment of balanced immune responses.

### Molecular mimicry and antigen cross-reactivity

The notion that immunity against microbes could confer protection against cancer traces back to early observations of tumor regression following bacterial infection [56]. Only recently has the mechanistic basis for this phenomenon been elucidated in the context of cancer immunotherapy, with molecular mimicry emerging as a key underlying process [57]. This mechanism proposes that T cells primed against antigens from commensal bacteria can cross-react with structurally or sequentially similar epitopes expressed by tumor cells, thereby providing a pre-existing pool of tumor-reactive T cells that can be mobilized upon ICB [57].

The theoretical foundation of molecular mimicry lies in T cell biology [35]. During thymic selection, developing T cells cannot completely eliminate all self-reactive clones, particularly those that may later encounter antigens derived from commensal microbes or tumor neoantigens [58]. In mesenteric lymph nodes, DCs sample commensal bacteria and present microbial peptides to naïve T cells [58]. When microbial epitopes share sufficient homology with tumor neoantigens, T cell receptors recognize both, generating cross-reactive T cells [59]. Evidence supporting this mechanism includes the identification of specific TCR clonotypes in immunotherapy responders that recognize both microbial epitopes and homologous tumor antigens [60].

Functionally, continuous exposure to commensal bacteria maintains cross-reactive T cells in a heightened memory-like state, creating a pre-primed anti-tumor immune force [59, 60]. Upon ICB, these T cells receive antigen-specific stimulation from tumor antigens, along with enhanced co-stimulatory signals, leading to rapid expansion and effector differentiation [60]. This mechanism may partly explain why some patients exhibit rapid and durable responses to ICIs, as their immune systems are effectively preconditioned by prior microbial exposure.

However, molecular mimicry is a double-edged sword. The same cross-reactivity that enhances anti-tumor immunity can also drive irAEs. Similar to mechanisms in autoimmune diseases, cross-reactivity between microbial and host antigens may underlie immunotherapy-induced colitis and other toxicities [61, 62]. Thus, the beneficial anti-tumor effects and detrimental side effects of ICIs may stem from the same microbiota-enhanced immune processes.

Although research on molecular mimicry in cancer immunotherapy is still emerging, it provides a compelling explanation for how the microbiota can supply antigen-specific effector T cells. This mechanism complements broader immunomodulatory effects mediated by microbial metabolites and intestinal barrier integrity, and may inform the development of microbial-based vaccines or personalized microbiota-targeted immunotherapies.

### The pivotal role of microbial metabolites in immune regulation

Beyond the direct recognition of microbial components, numerous small molecules produced by the gut microbiota serve as critical messengers in host-microbe crosstalk [63, 64]. These metabolites, either synthesized de novo or derived from host and dietary compounds, can disseminate systemically to modulate immune cell function and differentiation [65]. By regulating key pathways in both the TME and peripheral tissues, microbiota-derived metabolites influence T cell effector function, myeloid cell activity, epithelial integrity, and inflammatory tone [65–68]. Their structural and functional diversity allows them to exert both immunostimulatory and immunosuppressive effects, with the net outcome depending on the microbial composition and metabolic output of the gut ecosystem.

#### Short-chain fatty acids

**Fig. 3:**
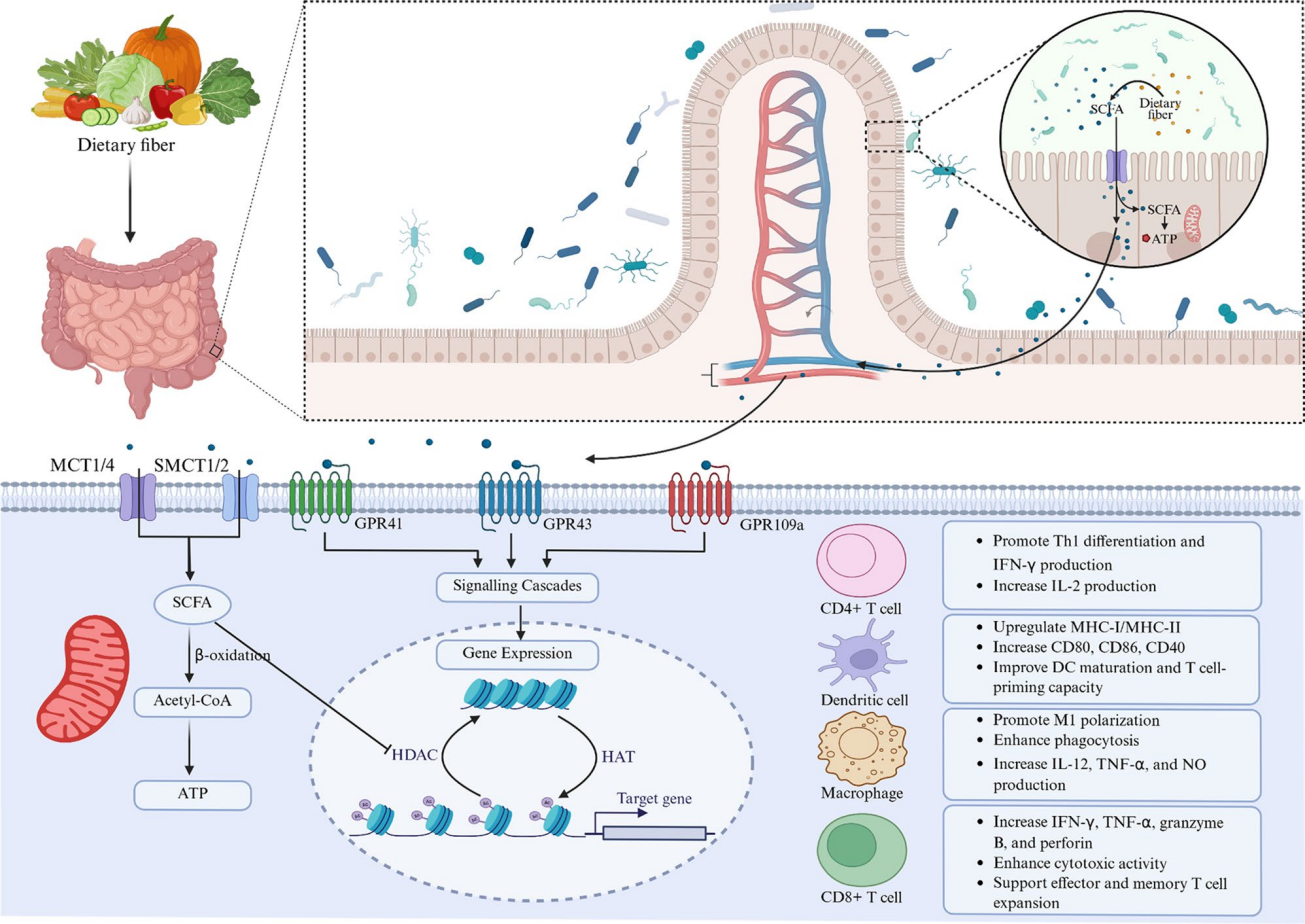
SCFAs modulate innate and adaptive immunity. They are derived from the bacterial fermentation of dietary carbohydrates in the colon. In addition to serving as an energy substrate, certain SCFAs can induce downstream gene transcription by inhibiting HDACs or activating GPCR-mediated signaling cascades, thereby regulating the effector responses of distinct innate and adaptive immune cells. SCFAs: Short-Chain Fatty Acids; GPR: G-protein coupled receptor; HDAC: histone deacetylase; HAT: histone acetyltransferase; SMCT: sodium-coupled monocarboxylate transporter; MCT: monocarboxylate transporter; TNF-α: Tumor Necrosis Factor-α;IL: Interleukin; CD: Cluster of Differentiation; MHC: Major Histocompatibility Complex; IFN-γ: interferon-γ. Original figure created with https://BioRender.com

SCFAs, including acetate, propionate, butyrate, isobutyrate, valerate, isovalerate and caproate, are microbial metabolites that modulate both tumor and immune cell biology. Beyond serving as energy substrates, SCFAs regulate gene expression, cell proliferation, and immune responses via G-protein-coupled receptor (GPCR) signaling and histone deacetylase (HDAC) inhibition (Fig. 3). These mechanisms enable SCFAs to coordinate cellular processes such as PCD and metabolic adaptation within the TME.

SCFAs engage GPCRs such as GPR41, GPR43, and GPR109A, which are widely expressed across immune cell populations, including DCs, macrophages, neutrophils, NK cells, and T cells [69, 70]. Engagement of these receptors triggers Gi/o-dependent signaling that reduces intracellular cAMP levels, modulates PKA activity, and consequently fine-tunes NF-κB, MAPK, and STAT pathways [71]. SCFAs regulate DCs maturation and antigen-presenting capacity in a context-dependent manner, often restraining excessive inflammatory activation while fine-tuning T-cell priming. In macrophages, GPCR activation promotes M1-like polarization while limiting M2-associated transcriptional programs [72]. In neutrophils, SCFAs regulate chemotaxis, activation thresholds, and cytokine responsiveness, contributing to coordinated innate immune activation in the TME [73].

Butyrate and propionate function as potent class I/II HDAC inhibitors that remodel chromatin accessibility in multiple immune cell types. In CD8 + T cells, SCFAs-mediated HDAC inhibition drives hyperacetylation of effector-gene loci, enhancing expression of IFN-γ, granzyme B, and perforin [74]. In DCs, HDAC inhibition promotes a more immunogenic phenotype by elevating antigen-presenting capacity and pro-inflammatory cytokine production [75]. In macrophages, SCFAs suppress transcriptional programs associated with pro-tumor M2 polarization while facilitating M1 reprogramming [76]. Furthermore, in CD4⁺ T cells, SCFAs-induced epigenetic modulation supports Foxp3 induction under appropriate cytokine conditions, allowing controlled Tregs differentiation to maintain immune balance in the TME [77, 78]. These chromatin-level changes provide durable transcriptional reinforcement of antitumor immunity.

SCFAs also serve as metabolic substrates that directly fuel immune cell energetics. Once internalized, SCFAs are converted into acetyl-CoA and oxidized in the TCA cycle, thereby supporting oxidative phosphorylation (OXPHOS) in effector and memory T cells as well as NK cells [79]. Enhanced OXPHOS promotes long-term survival, proliferation, and sustained cytotoxicity, particularly within the nutrient-limited and hypoxic TME. SCFAs-mediated HDAC inhibition additionally upregulates genes involved in glycolysis and fatty-acid oxidation, improving metabolic flexibility and allowing immune cells to maintain effector function under metabolic stress [80, 81]. This metabolic support is crucial for optimizing cytotoxic activity and memory formation during antitumor responses.

The immunomodulatory actions of SCFAs arise from the integration of rapid receptor signaling, longer-lasting epigenetic remodeling, and sustained metabolic support [69, 82]. GPCR pathways rapidly modulate cytokine release and immune cell trafficking [83]; HDAC inhibition stabilizes transcriptional programs that enhance effector function [84]; and SCFAs-derived metabolic fueling promotes persistence and memory formation [85]. Together, these coordinated mechanisms strengthen antigen presentation, cytotoxicity, memory T-cell development, and calibrated inflammatory responses, collectively reinforcing antitumor immunity within the TME. These insights highlight the therapeutic potential of targeting the microbiota-SCFAs-immune axis to improve the efficacy of cancer immunotherapy and provide a translational framework for microbiota-driven metabolic interventions.

#### Tryptophan derivatives

**Fig. 4:**
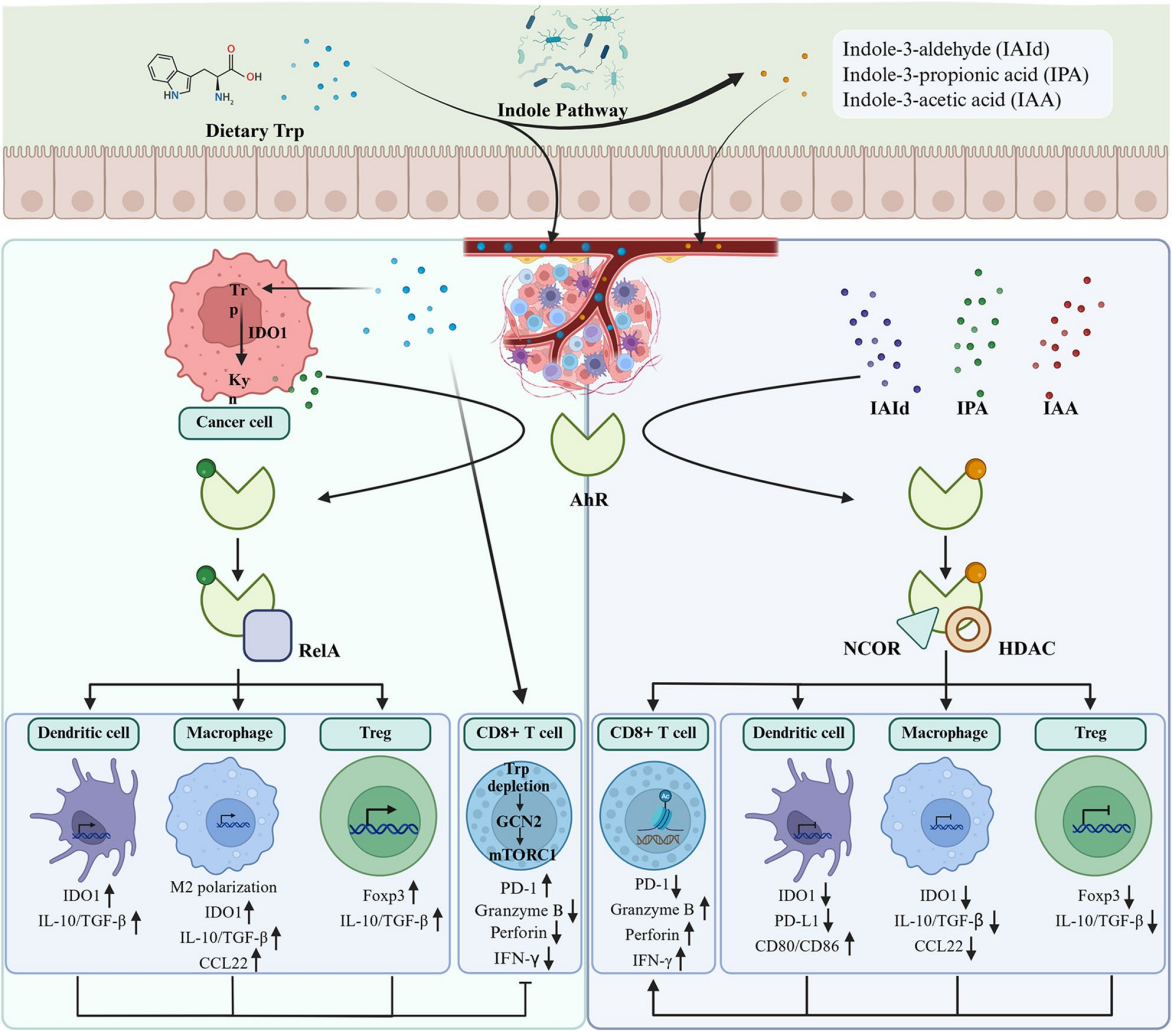
Dietary tryptophan metabolites regulate tumor immunity through the kynurenine and indole pathways. In the TME, tryptophan is preferentially catabolized via the kynurenine pathway by tumor cells, leading to tryptophan depletion and kynurenine accumulation, which suppress CD8 + T cell proliferation and effector function through activation of the GCN2-mTORC1 stress response and AhR-dependent tolerogenic signaling. In contrast, certain gut microbiota-derived indole metabolites enter systemic circulation and elicit a ligand-biased AhR signaling program that restrains tolerogenic dendritic cell and macrophage programming, limits Tregs-mediated immunosuppression, and preserves CD8 + T cell metabolic fitness and cytotoxic activity, thereby supporting antitumor immunity. Trp: tryptophan; Kyn: kynurenine; AhR: aryl hydrocarbon receptor; Foxp3: forkhead box P3; IDO1: indoleamine 2,3-dioxygenase 1; STAT1: signal transducer and activator of transcription 1; RelA: v-rel avian reticuloendotheliosis viral oncogene homolog A; NCOR: nuclear receptor corepressor; HDAC: histone deacetylase; Treg: regulatory T cell; IL: interleukin; CD: cluster of differentiation; TGF-β: Transforming Growth Factor-β; CCL: C-C motif chemokine ligand; PD-1: Programmed Cell Death Protein 1. Original figure created with https://BioRender.com

Tryptophan is an essential amino acid, primarily obtained from dietary intake. Beyond its role as a building block for protein synthesis, tryptophan serves as a substrate for multiple metabolic pathways that have significant implications for immune regulation and tumor biology [86, 87]. Emerging evidence indicates that the gut microbiota acts as a key regulator of tryptophan metabolism, influencing substrate partitioning, metabolic flux, and host enzymatic activity [88–90]. Through these interactions, the microbiota-tryptophan metabolic axis critically shapes the balance between immunostimulatory and immunosuppressive signals within the TME (Fig. 4).

Mechanistically, dietary tryptophan undergoes metabolic partitioning into multiple pathways, generating diverse metabolites that differentially regulate immune responses in a context-dependent manner. In the intestinal lumen, a fraction of tryptophan is metabolized by gut microbiota into indole and its derivatives, whereas the absorbed pool is further processed in host cells through the kynurenine and serotonin pathways [86]. Both kynurenine and microbiota-derived indole metabolites act as ligands for the aryl hydrocarbon receptor (AhR), but can induce distinct and context-dependent transcriptional programs depending on ligand structure and cellular environment. Binding of kynurenine to AhR is frequently associated with immunosuppressive gene expression, including IL10 and FOXP3, thereby promoting Tregs differentiation and immune tolerance [91–93]. In contrast, indole-derived metabolites can differentially modulate AhR activity, leading to diverse transcriptional outcomes that may either support barrier integrity and immune homeostasis or fine-tune inflammatory responses [94, 95]. This ligand-dependent competition for AhR establishes a metabolic checkpoint that critically shapes immune fate decisions within the TME.

Notably, indole-derived metabolites exert context-dependent and sometimes opposing effects on tumor immunity. In certain tumor models, indole-3-aldehyde (I3A) has been shown to enhance antitumor immunity by increasing tumor immunogenicity and promoting CTL responses [94]. These effects have been associated with multiple mechanisms, including modulation of antigen presentation, inflammatory cytokine signaling, and oncogenic pathways, although the precise molecular circuitry appears to vary across experimental contexts [94]. Conversely, some indole metabolites can activate AhR signaling in tumor-associated macrophages (TAMs), driving their polarization toward an immunosuppressive M2-like phenotype characterized by elevated secretion of IL-10 and TGF-β, ultimately dampening CD8⁺ T cell cytotoxicity [96]. The dual immunological roles of indole metabolites likely reflect differences in ligand affinity, cellular context, receptor cofactor availability, and metabolic concentrations within the TME.

Beyond direct indole production, the gut microbiota also exerts indirect yet potent control over the kynurenine pathway [97, 98]. The host kynurenine pathway is initiated by the rate-limiting enzymes indoleamine 2,3-dioxygenase (IDO1/IDO2) and tryptophan 2,3-dioxygenase (TDO), both of which are frequently upregulated in tumors and associated with immune evasion [99–102]. Specific commensal bacteria can suppress kynurenine accumulation by downregulating IDO1 expression or activity. For example, Lactobacillus gallinarum-derived indole-3-carboxylic acid reduces intratumoral kynurenine levels, enhances responsiveness to immune checkpoint blockade, and competitively antagonizes kynurenine-AhR signaling in CD4⁺ T cells, thereby limiting Tregs differentiation [97]. Through these mechanisms, the gut microbiota reshapes the kynurenine landscape to favor antitumor immunity.

Collectively, these findings highlight the gut microbiota as a central orchestrator of tumor immunity through its dual regulation of the indole and kynurenine pathways of tryptophan metabolism. By controlling ligand availability and competitive AhR signaling, microbial communities fine-tune the transcriptional balance between immune activation and immune tolerance within the TME. Targeting this microbiota-tryptophan-AhR axis therefore represents a promising strategy to reprogram tumor immunity and enhance the efficacy of cancer immunotherapies.

#### Bile acids

**Fig. 5:**
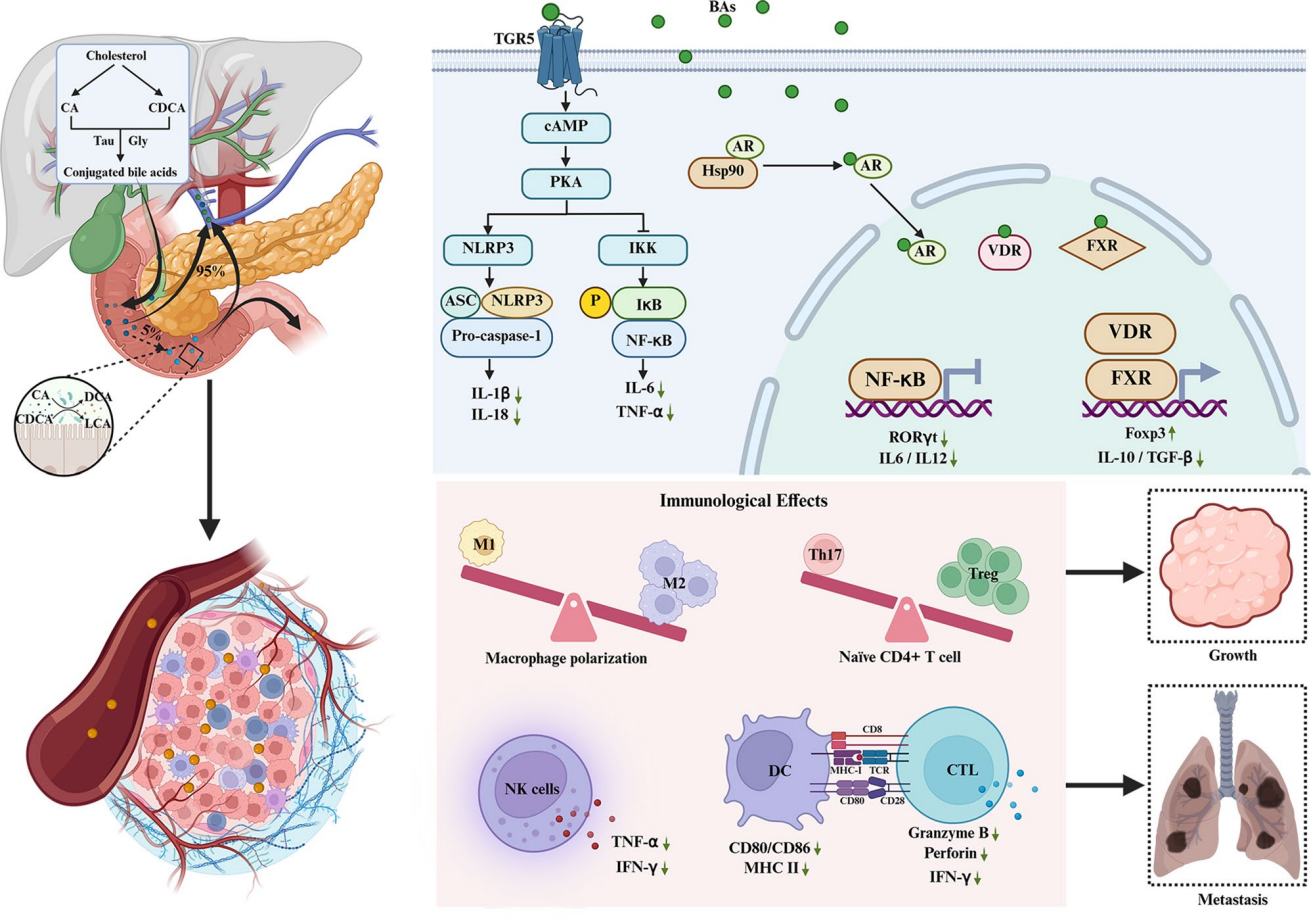
Gut microbiota modulates tumor immunity through bile acid metabolism. Primary bile acids are synthesized in the liver and secreted into the intestine, where gut microbiota convert them into secondary bile acids. Within the TME, bile acids modulate inflammatory signaling pathways, including NF-κB, primarily through canonical receptors such as the membrane receptor TGR5 and the nuclear receptor FXR. In addition, specific microbiota-derived bile acid metabolites can interact with non-canonical nuclear receptors, including VDR and the AR, in a context-dependent manner. These effects collectively lead to enhanced Tregs differentiation, M2 macrophage polarization, and impaired dendritic cell antigen-presenting function, thereby contributing to the establishment of an immunosuppressive TME. CA: cholic acid; CDCA: chenodeoxycholic acid; LCA: lithocholic acid; DCA: deoxycholic acid; Tau: taurine; Gly: glycine; BAs: bile acids; TGR5: G-protein-coupled bile acid receptor 1; cAMP: cyclic adenosine monophosphate; PKA: Protein kinase A; NLRP3: NOD-, LRR- and pyrin domain-containing protein 3; ASC: apoptosis-associated speck-like protein containing a CARD; IKK: IκB kinase; AR: androgen receptor; VDR: vitamin D receptor; FXR: farnesoid X receptor; Foxp3: forkhead box P3; RORγt: retinoic acid-related orphan receptor gamma t; TCR: T cell receptor; Tregs: regulatory T cell; IL: interleukin; CD: cluster of differentiation; IFN-γ: interferon-γ; TNF-α: tumor necrosis factor-α. Original figure created with https://BioRender.com

Bile acids, synthesized from cholesterol in the liver, are essential metabolites that facilitate lipid absorption and maintain metabolic and immune homeostasis [103]. Primary bile acids, mainly cholic acid and chenodeoxycholic acid, are secreted into the intestine, where they undergo extensive microbial transformation in the distal ileum and colon. Gut bacterial enzymes catalyze these transformations, generating a diverse array of secondary bile acids, including deoxycholic acid (DCA), lithocholic acid (LCA), and ursodeoxycholic acid (UDCA) [104]. This microbial processing significantly diversifies the bile acid pool, converting primary bile acids into potent bioactive signaling molecules that profoundly influence host immunity (Fig. 5).

Mechanistically, bile acids function as versatile signaling molecules by engaging both membrane-bound and nuclear receptors. The membrane receptor G protein-coupled receptor 1 (GPBAR1/TGR5) senses extracellular bile acids [105], whereas the nuclear receptor farnesoid X receptor (FXR) mediates transcriptional programs in immune and non-immune cells [106, 107]. Beyond these canonical pathways, bile acids, particularly microbiota-derived derivatives, can also modulate additional nuclear receptors, such as the androgen receptor (AR) and vitamin D receptor (VDR) [106, 108, 109]. Through these interactions, bile acids regulate multiple downstream signaling pathways, including MAPK/ERK, STAT3, NF-κB, and JNK/AP-1, thereby influencing the transcription of cytokines, chemokines, and other immunoregulatory molecules [110–112]. These signaling pathways shape immune cell function within the TME, directing TAM polarization [105, 113], modulating DCs antigen-presenting capacity [114, 115], and influencing the differentiation and activity of Tregs [116–119]. Together, this receptor-signaling network establishes a bile acid-dependent regulatory axis linking gut microbial metabolism to the modulation of immune cell states within the TME.

Functionally, bile acids exert context-dependent, bidirectional effects on tumor immunity. On the one hand, certain bile acid species have been reported to enhance NK cell proliferation and cytotoxic activity, potentially through activation of MAPK/ERK and STAT3 signaling pathways [120]. Additionally, the secondary bile acid 3-oxo-Δ4,6-lithocholic acid acts as an AR antagonist, preventing its nuclear translocation and transcriptional activity, which augments CD8⁺ T cell-mediated antitumor immunity [121]. UDCA further enhances antitumor immunity by promoting TGF-β degradation, thereby restraining Tregs differentiation and activation [122]. On the other hand, bile acids can foster an immunosuppressive TME. Elevated bile acid levels drive M2 polarization of TAMs through FXR-dependent signaling, facilitating tumor progression [105, 113]. Bile acids can also activate the JNK/AP-1 pathway in tumor cells, leading to PD-L1 upregulation and inhibition of T cell cytotoxicity [123]. The impact on T cells is additionally influenced by bile acid species: primary bile acids may induce oxidative stress and T cell exhaustion or apoptosis, whereas LCA suppresses T cell function via endoplasmic reticulum stress, and UDCA preserves T cell viability [124].

Collectively, this duality reflects bile acid heterogeneity, receptor specificity and the cellular and pathophysiological context within the TME. These factors ultimately determine whether bile acid signaling promotes immune activation or immunosuppression in cancer.

#### Other microbiota-derived metabolites

Beyond the well-established roles of short-chain fatty acids, tryptophan derivatives, and bile acids, emerging evidence indicates that the gut microbiota produces a wide array of additional metabolites that critically modulate host immunity and shape the TME. These microbiota-derived molecules influence immune cell activation, differentiation, and effector functions through distinct yet interconnected mechanisms, thereby fine-tuning anti-tumor immunity.

Adenosine, a purine nucleoside, can be generated by host cells and, to a lesser extent, by the metabolic activities of specific gut microbes through nucleotide degradation pathways. Within the TME, extracellular adenosine accumulates and exerts potent immunosuppressive effects primarily via the enzymatic conversion of extracellular ATP through the CD39/CD73 axis, particularly under hypoxic conditions, and engages A2A and A2B receptors on immune cells [125, 126]. Activation of these receptors inhibits the cytotoxic functions of CD8^+^ T cells and NK cells, while promoting the expansion and function of Tregs and myeloid-derived suppressor cells (MDSCs) [127–129]. Furthermore, adenosine signaling impairs DCs maturation and alters cytokine production, thereby indirectly hampering the priming of effective T cell responses [130].

Inosine, a deamination product of adenosine, is another nucleoside that can be generated by gut microbiota and host metabolism, with context-dependent roles in anti-tumor immunity. Certain bacterial species such as Akkermansia muciniphila [131] and Bifidobacterium pseudolongum [132, 133], are proficient inosine producers. Inosine has been shown to support T cell function under glucose-restricted conditions by serving as an alternative carbon source, thereby promoting CD8⁺ T cell proliferation and effector activity [134]. Beyond metabolic support, inosine and its derivative isoprinosine have been reported to enhance tumor immunogenicity and improve the efficacy of ICB [135]. Additionally, recent studies suggest that inosine can promote the stem-like characteristics and persistence of CAR-T cells through metabolic and epigenetic reprogramming, thereby enhancing their anti-tumor efficacy in certain contexts [136].

Polyamines, such as putrescine, spermidine, and spermine, are low-molecular-weight aliphatic compounds essential for cell proliferation and differentiation. Gut microbes, particularly Bacteroides species, are major producers of polyamines in the intestine [137]. Within the TME, polyamine metabolic reprogramming is a hallmark of cancer, contributing to an immunosuppressive milieu. Tumor cells upregulate biosynthetic enzymes like ornithine decarboxylase, while microbial-derived polyamines further augment the pool [138]. Elevated polyamine levels drive macrophage polarization toward an immunosuppressive M2 phenotype and directly suppress T cell activation and proliferation [139]. Furthermore, polyamines can activate protein kinase CK2, enhancing the suppressive capacity of Tregs [140]. They also increase tumor cell intrinsic tolerance to immune attack [141]. Thus, both host- and microbiota-derived polyamines serve as crucial mediators linking cellular metabolism to immune evasion, positioning polyamine metabolism as a promising therapeutic target.

Collectively, the gut microbiota regulates tumor immunity through a vast and diverse repertoire of metabolites. Elucidating these intricate metabolite-mediated mechanisms profoundly expands our understanding of the microbiota-tumor-immune axis and opens new avenues for therapeutic interventions aimed at bolstering anti-tumor immunity by targeting the gut microbiome.

## Therapeutic intervention of gut microbiota in cancer immunotherapy

**Fig. 6:**
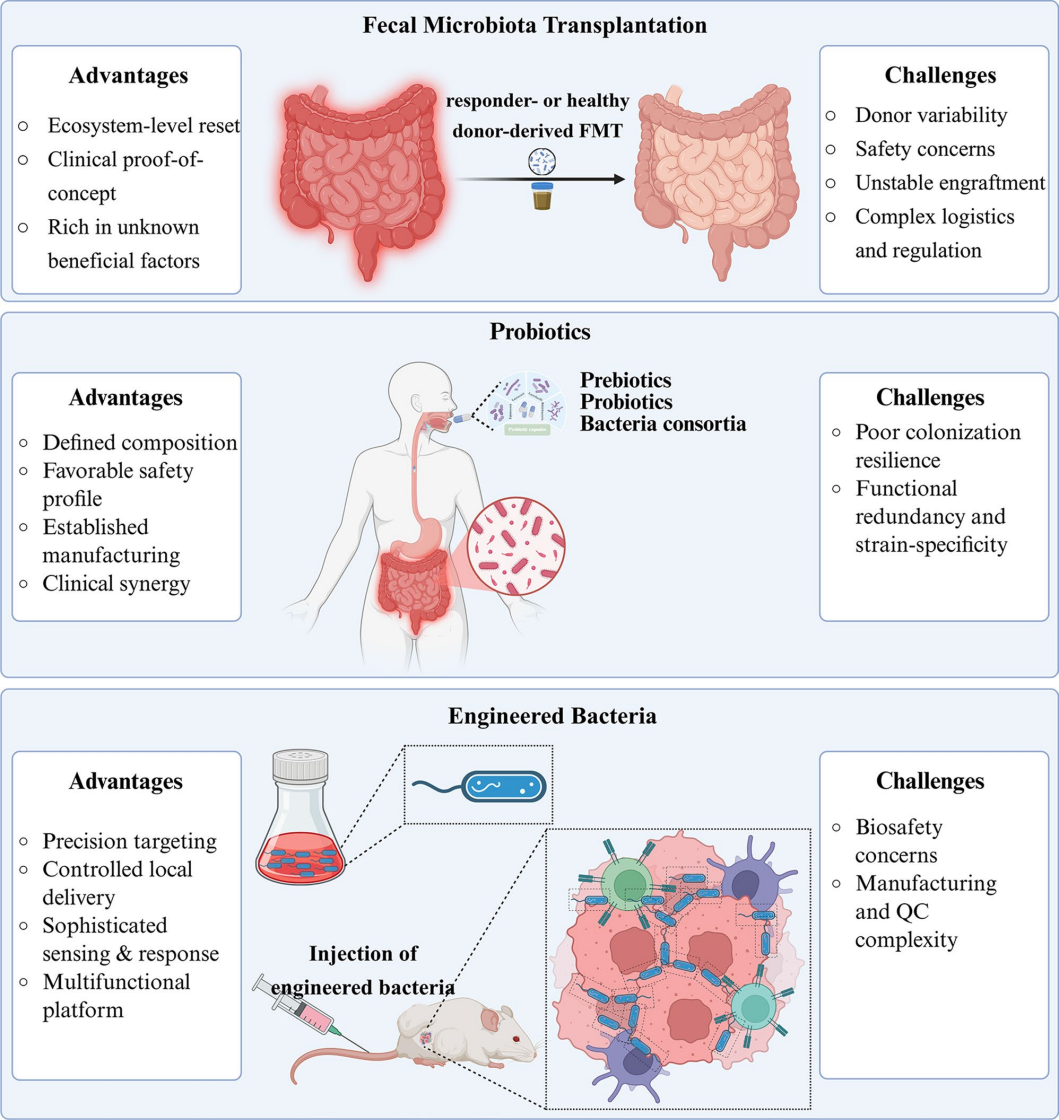
Different therapeutic interventions exhibit specific advantages and limitations. Once applied, these strategies can reshape the microbial composition, restore epithelial barrier function, modulate inflammatory signaling, and enhance immune surveillance. However, their therapeutic efficacy is often influenced by inter-individual variability, heterogeneity of the microbiota, efficiency of delivery, and potential immune-related adverse effects. FMT: fecal microbiota transplantation; QC: quality control. Original figure created with https://BioRender.com

Building upon the mechanistic understanding of how the gut microbiota influences anti-tumor immunity, a spectrum of intervention strategies has emerged, ranging from holistic ecosystem resets to precise molecular engineering. This evolution reflects the transition from empirical approaches toward rationally designed, mechanism-driven interventions, each with its own advantages and limitations (Fig. 6).

### FMT: ecosystem reset and its inherent limitations

Among these strategies, FMT has emerged as a particularly promising yet complex intervention. This therapeutic modality involves transferring processed fecal microbiota from healthy or treatment-responsive donors into recipients, representing a paradigm shift from tumor-centric therapies toward systemic modulation of host-microbiome interactions that shape anti-tumor immunity.

A series of clinical studies has demonstrated the dual potential of FMT in cancer immunotherapy: overcoming anti-PD-1 resistance and mitigating treatment-related complications (Table 1). Two phase I clinical trials established proof-of-concept by transplanting gut microbiota from anti-PD-1 responders into ICIs-refractory melanoma patients, successfully restoring anti-tumor immunity and inducing objective clinical responses in a significant subset (20–30%) of patients [142, 143]. Subsequent investigation showed that the addition of FMT to ICIs was well tolerated and may contribute to overcoming primary resistance to ICIs [31]. This pivotal shift in donor selection, employing FMT from healthy donors rather than ICIs responders, offers a practical pathway toward broader clinical application. While head-to-head comparisons are lacking, healthy-donor FMT theoretically provides higher microbial diversity, greater abundance of beneficial taxa, and the potential for personalized donor-recipient matching. Conversely, feces from treatment responders may enrich microbial communities specifically associated with sustained ICI responses.

**Table 1:** Clinical applications of FMT in cancer: efficacy enhancement and toxicity management

Reference	Year	Intervention	Primary Outcome	Study Type
Davar et al. [142]	2021	Responder-derived FMT with anti-PD-1 administered to 15 patients with PD-1-refractory melanoma.	The clinical benefit was observed in 6/15 patients.	Phase I clinical trial
Baruch et al. [143]	2021	Responder-derived FMT with anti-PD-1 administered to 10 patients with PD-1-refractory melanoma.	The clinical response was observed in 3/10 patients, including 2 PR and 1 CR.	Phase I clinical trial
Routy et al. [31]	2023	Healthy donor FMT with the PD-1 inhibitors administered to 20 previously untreated patients with advanced melanoma.	25% experienced grade 3 irAEs. The ORR was 65%, including 20% CR.	Phase I clinical trial
Hadi et al. [144]	2025	Healthy donor FMT with PD-1 inhibitors administered to 20 previously untreated patients with advanced melanoma.	The mPFS was 29.6 months, mOS 52.8 months. The 1, 2, and 3 years estimated survival rates were 95%, 74% and 53%.	Phase I clinical trial
Kim et al. [32]	2024	Responder-derived FMT with anti-PD-1 therapy administered to 13 patients with PD-1-refractory advanced solid tumors.	The ORR was 7.7% (PR in 1 of 13 patients)	Phase I clinical trial
Glitza et al. [145]	2024	Ser-401 with PD-1 inhibitors administered to 14 patients with ICB-naïve metastatic melanoma.	The ORR was 25% in the SER-401 arm 67% in the placebo arm.	Phase I clinical trial
Halsey et al. [146]	2023	Healthy donor FMT administered to in 12 patients with refractory IMC	The clinical remission was 92%	Phase I clinical trial
Elkrief et al. [147]	2024	Healthy donor FMT administered to in 5 patients with refractory IMC	The clinical remission was 80%	Phase I clinical trial
Zhao et al. [148]	2023	FMT with plus tislelizumab and fruquintinib administered to 20 patients with refractory MSS metastatic colorectal cancer	mPFS was 9.6 months. mOS was 13.7 months. ORR was 20%	Phase II clinical trial

FMT: fecal microbiota transplantation; ICIs: immune checkpoint inhibitors; PD-1: programmed cell death 1; PR: partial response; CR: complete response; irAEs: immune-related adverse events; ORR: objective response rate; mPFS: median progression-free survival; mOS: median overall survival; MSS: microsatellite stable; SER-401: an orally delivered Firmicutes-enriched spore formulation; IMC: immune-mediated colitis

The evidence supporting FMT in oncology is steadily growing. A prospective single-arm trial conducted in Korea demonstrated that FMT from donors exhibiting durable responses to anti-PD-1 therapy could restore immunotherapy efficacy in patients with advanced solid tumors [32]. Microbial profiling identified Prevotella merdae Immunoactis as an immunostimulatory strain enhancing T cell activity, whereas Lactobacillus salivarius and Bacteroides plebeius were associated with inhibitory effects, highlighting the functional specificity of gut microbiota in clinical outcomes. In parallel, a phase II trial reported that the combination of FMT with tislelizumab and fruquintinib elicited promising clinical activity and a manageable safety profile in patients with refractory microsatellite-stable metastatic colorectal cancer, a population historically resistant to immunotherapy [148]. Moreover, emerging evidence suggests that FMT may attenuate irAEs, including immunotherapy-induced colitis [146, 147], providing the dual benefits of enhanced anti-tumor immunity and reduced treatment-associated toxicity. Collectively, these studies establish microbiota modulation as a viable next-generation immunotherapy strategy, warranting further mechanistic and translational investigation.

Despite these encouraging findings, the core challenge of FMT lies in its inherent “black box” nature. First, the high heterogeneity of donor material challenges standardization and predictability. Variability in microbial composition, metabolic function, and immunomodulatory potential contributes to inconsistent clinical outcomes. This reality necessitates a critical evolution in donor selection, where the existing framework must shift from a predominantly safety-oriented screening paradigm toward efficacy-driven matching strategies. Current guidelines prioritize safety by excluding pathogens and donors with disrupted gut ecosystems [149, 150], but rarely incorporate systematic evaluation of therapeutic potential. Evidence increasingly links FMT success to donor characteristics such as microbial alpha-diversity, ecological compatibility with the recipient, and abundance of key functional taxa [151, 152]. Furthermore, the donor’s virome and mycobiome may modulate efficacy [153], though these factors are often overlooked. While the “super-donor” concept initially focused on donor traits, recent data suggest recipient factors may be more decisive, emphasizing the importance of personalized matching [154]. These considerations, along with persistent safety concerns regarding undetected pathogens [155] or adverse metabolic consequences [156], highlight the need for comprehensive donor evaluation systems that optimize both safety and therapeutic potential.

Second, ecological complexity limits stable engraftment. The resident microbiota exerts “colonization resistance”, impeding integration of transplanted communities [157]. Strategies such as antibiotic preconditioning can enhance initial colonization but rarely ensure long-term stability [151, 158]. Emerging evidence emphasizes the dominant role of recipient factors such as genetic background, immune status, and baseline microbiota, in post-transplant microbial reconstruction [151]. Disease-specific patterns exist: lower baseline diversity often predicts better engraftment in certain conditions [159, 160], whereas the opposite is observed in ulcerative colitis and irritable bowel syndrome [161]. Additionally, donor-recipient compatibility shows nuanced effects, where subspecies-level genomic congruence predicts strain transfer efficiency [160], yet lower overall similarity can sometimes yield superior clinical outcomes [162]. These observations underscore the need for individualized, recipient-focused approaches rather than universal donor criteria.

Third, intertwined safety concerns and regulatory hurdles continue to pose substantial obstacles to the widespread clinical adoption of FMT. Although serious adverse events remain relatively rare, the potential for adverse outcomes such as the inadvertent transmission of undetected pathogens, the initiation of immune-mediated complications, and the emergence of unpredictable long-term health effects, cannot be entirely ruled out [163, 164]. These safety apprehensions are further magnified by the absence of universally standardized manufacturing processes and quality assurance systems, which together hinder the transition of FMT from a laboratory practice to a reproducible, pharmaceutical-grade product. This technical and regulatory uncertainty becomes particularly evident as FMT seeks formal approval beyond the well-established indication of recurrent Clostridioides difficile infection, especially within oncology, where rigorous clinical validation and optimized procedural protocols are urgently needed. The existing technical landscape remains heterogeneous and fragmented, with different administration routes such as colonoscopy, nasoenteric infusion, and oral encapsulation, each offering distinct advantages and drawbacks in terms of delivery precision, invasiveness, microbial viability, and patient compliance [165, 166]. Furthermore, the lack of uniform preparation and preservation methods complicates reproducibility and scalability. Conventional frozen formulations require a stringent − 80 °C cold chain, which restricts logistical flexibility and limits global accessibility. In contrast, lyophilized or “off-the-shelf” FMT products have recently emerged as promising alternatives, improving stability, storage feasibility, and potential commercialization [167, 168]. Compounding these technical limitations, divergent regulatory frameworks across jurisdictions further impede harmonization [169]. Such discrepancies create barriers to international standardization, obstructing multi-center clinical trials and delaying the global implementation of FMT as a clinically accepted, regulated therapeutic platform.

In conclusion, while FMT has established itself as a powerful proof-of-concept, demonstrating the profound influence of the gut microbiome on cancer immunotherapy outcomes, its future role in oncology depends on transforming it from an empirical procedure into a precision therapeutic modality. Achieving this transformation requires developing sophisticated donor selection strategies based on immune-relevant functional criteria rather than convenience or availability. It also necessitates the implementation of ecological pre-conditioning approaches to promote consistent and durable microbial engraftment. Crucially, FMT products must be produced under pharmaceutical-grade standards, incorporating rigorous quality control, standardized manufacturing processes, and validated potency assays. By systematically addressing these challenges through collaborative research and interdisciplinary innovation, the full therapeutic potential of microbial ecosystem transfer can be more predictably realized, ultimately benefiting the increasingly heterogeneous population of cancer patients undergoing immunotherapy. The transition from an ill-defined “black box” to a function-driven precision intervention is already underway, and this evolution is expected to significantly expand the strategic landscape of cancer therapy in the coming years.

### Probiotics: functional potential with precision constraints

Probiotics, defined as live microorganisms that confer a health benefit to the host when administered in adequate amounts, represent a key component of the human microbiome with established roles in maintaining systemic homeostasis [170]. A growing body of evidence demonstrates that probiotic supplementation can ameliorate a spectrum of diseases, including antibiotic-associated diarrhea [171], inflammatory bowel disease [172], metabolic disorders [173]. Recently, probiotics supplementation has attracted increasing attention in cancer prevention and therapy due to its accessibility and promising immunomodulatory potential [174–177].

The primary antitumor mechanism of probiotics lies in their ability to prevent tumor initiation by modulating gut microecological balance. Specifically: (i) they enhance intestinal barrier integrity by stimulating mucus secretion and strengthening tight junctions between epithelial cells, effectively preventing pathogen translocation and thereby reducing systemic chronic inflammation [172, 178, 179], which is a well-established tumor-promoting factor; (ii) they provide colonization resistance by competing with pathogens for nutrients and ecological niches, as well as producing bactericidal metabolites, directly inhibiting the growth of potential pathobionts and maintaining a benign microbial equilibrium [180, 181]; (iii) certain probiotics strains can enhance the host’s antioxidant capacity by producing superoxide dismutase or modulating the host’s own antioxidant signaling pathways such as the Nrf2 pathway, thereby scavenging reactive oxygen species (ROS) and mitigating DNA damage from oxidative stress [181, 182], preventing tumor initiation at its root.

Beyond the above mechanisms, a central mechanism by which probiotics exert direct antitumor effects is through the modulation of the host’s systemic immune responses [183]. Probiotic metabolites, particularly SCFAs, play a pivotal role in shaping the immune landscape both within the intestine and systemically [69, 184]. For example, butyrate functions as a potent HDAC inhibitor that epigenetically reprograms immune cell function [185, 186]. In parallel, certain tryptophan-derived indole metabolites produced by probiotic species engage the AhR pathway, promoting IFN-γ-producing CD8 + T cells [94, 97], which contributes to enhanced antitumor immunity. Moreover, specific probiotic strains, such as Bifidobacterium species, have been shown to enhance antigen presentation by DCs and bolster the cytotoxic activity of CD8 + T cells, leading to improved immune surveillance and tumor control [21, 187].

Given the strong immunomodulatory activity of gut microbes, increasing evidence supports combining probiotics with ICIs to enhance anticancer efficacy. Numerous preclinical studies have explored the mechanisms underlying this synergistic effect [176, 188–190]. For example, Clostridium butyricum augments anti-PD-1 activity in AOM/DSS-induced and germ-free CRC models through its surface protein SecD binding to GRP78 on tumor cells, thereby reducing IL-6 secretion and subsequently alleviating IL-6-mediated suppression of CTL and induction of TAMs [176]. In another mouse tumor model, oral administration of the exopolysaccharide produced by Lactobacillus delbrueckii enhanced the efficacy of anti-CTLA-4 and anti-PD-1 therapies against CCL20-expressing tumors by increasing the infiltration of IFN-γ-producing CCR6⁺ CD8⁺ T cells [188]. These findings are further supported by clinical data (Table 2). For instance, a phase I clinical study demonstrated that in patients with metastatic renal cell carcinoma, the addition of the probiotics CBM588 to a regimen of nivolumab plus ipilimumab extended the median PFS from 2.5 to 12.7 months compared to immunotherapy alone [191]. Consistently, a subsequent report from the same research group indicated that adding CBM588 to a combination of nivolumab and cabozantinib increased the overall response rate from 20% to 74% and improved the 6-month PFS rate from 60% to 84% [192]. These results underscore the potential of specific probiotics to deliver consistent clinical benefits across different therapeutic regimens.

**Table 2:** Clinical studies on oral probiotic combined with ICIs in cancer

Reference	Year	Study Type	Cancer Type	Sample Size	Intervention	Primary outcome	Safety Profile
Dizman [191]	2022	Phase I clinical trial	Treatment-naive mRCC	30	Dual immune therapy (Nivo and ipilimumab) with or without daily oral CBM588	PFS: (CBM588 + ICIs): 12.7 months vs. ICIs alone: 2.5 months (*P* = 0.001)	No significant toxicity difference
Ebrahimi [192]	2024	Phase I clinical trial	mRCC	30	Cabo + Nivo with or without daily oral CBM588	6-month PFS rate: CBM588 + Cabo + Nivo): 84% vs. Cabo+Nivo alone: 60%; ORR (CBM588 + Cabo + Nivo): 74% vs. Cabo + Nivo alone: 20% (*P* = 0.01)	No significant toxicity difference
Tomita [193]	2022	Retrospective Study	Advanced or recurrent NSCLC	72	ICIs and PPI therapy with or without oral CBM588 (administered within 6 months prior to ICIs)	PFS: (PPI + ICIs + CBM588): 250 days vs. (PPI + ICIs): 88 days (*P* = 0.03)	/
Tomita [194]	2020	Retrospective Study	Advanced NSCLC	118	ICIs with or without oral CBM588 (administered within 6 months prior to ICIs)	Significantly prolonged PFS and OS	/
Tomita [195]	2023	Retrospective Study	metastatic NSCLC	106	Chemoimmunotherapy with or without oral CBM588	PFS: (ICIs + chemotherapy + CBM588): 9 months vs. (ICIs + chemotherapy): 5 months	/
Spreafico [196]	2023	Phase I clinical trial	Advanced solid tumors	39	ICIs with or without MET4	Clinical benefit: (ICIs + MET4) 53% (9/17) vs. (ICIs) 20% (1/5)	No MET4-related grade ≥ 3 AEs

ICIs: immune checkpoint inhibitors; mRCC: metastatic renal cell carcinoma; NSCLC: non-small cell lung cancer; PPI: proton pump inhibitor; PFS: progression-free survival; OS: overall survival; Nivo: nivolumab; Cabo: cabozantinib; MET4: microbial ecosystem therapeutic 4 (consortia of 30 bacterial strains); CBM588: clostridium butyricum MIYAIRI 588 (bifidogenic live bacterial product)

However, it must be cautiously acknowledged that probiotics are not a panacea, and their use in some solid tumors may even negatively impact immunotherapy. In vitro experiments have shown that among several commonly used lactic acid bacteria, Lactobacillus reuteri and Lactobacillus murinus produce indole-3-acetic acid, which activates the AhR on TAMs, thereby promoting pancreatic tumor progression and undermining the efficacy of ICB [96]. Similarly, a pivotal clinical study in patients with advanced melanoma indicated that self-administered probiotics were not associated with improved PFS [197]. Subsequent mouse experiments further demonstrated that supplementation with Lactobacillus rhamnosus or Bifidobacterium longum reduced intra-tumoral IFN-γ⁺ CD8⁺ T-cell infiltration and impaired the efficacy of anti-PD-L1 therapy, while promoting tumor growth [197]. These findings highlight that probiotics interventions should follow a personalized and precision-based approach rather than a universal strategy.

Effective probiotic-based modulation of tumor immunity depends on the ability of administered bacteria to survive gastrointestinal stress and retain functional activity. However, most commercially available probiotic formulations fall short of therapeutic requirements. A major hurdle is that many orally administered probiotics fail to survive the harsh conditions of the gastrointestinal tract, including gastric acid, bile salts, and digestive enzymes [198]. Furthermore, probiotic formulations are highly susceptible to environmental stressors during production, storage, and transportation, including temperature, humidity, and oxidative stress [199]. Such stressors often cause a significant loss of viable bacteria before administration, reducing the effective dose and therapeutic potential. To overcome these challenges and maximize the antitumor efficacy of probiotics, several innovative strategies are being explored.

**Advanced oral delivery systems for targeted release and colonization**: To increase the number of probiotics that reach and colonize the gut, various oral delivery systems have been developed, including polymeric microcapsules, lipid-based carriers, and biopolymer-derived matrices with pH-responsive and mucoadhesive properties [181, 200–205]. An effective microencapsulation system should fulfill several critical functions: (i) maintaining the stability of probiotics during storage; (ii) protecting them from adverse gastrointestinal fluids upon ingestion; and (iii) ensuring the controlled release of viable probiotic cells at the designated site in the gastrointestinal tract. For example, encapsulation using biomaterials such as alginate-chitosan double layers has proven effective in shielding bacteria from gastric acid, with the capsule dissolving specifically in the neutral pH of the intestines to facilitate localized release [200]. Similarly, liposome-based and pectin/zein-based microcapsules have been designed to provide robust protection and enhance mucosal adhesion in the colon, thereby significantly improving bacterial survival and persistence in vivo [201].

#### Rational combination with prebiotics and dietary interventions

The combination of probiotics with prebiotics, a strategy known as synbiotics, represents a powerful approach to support probiotic function [206–208]. Prebiotics, such as inulin [209] and fructooligosaccharides [210], serve as specialized nutrients that selectively stimulate the growth and metabolic activity of beneficial probiotic strains. This not only improves the colonization and persistence of administered probiotics but also promotes the production of beneficial metabolites, which can directly enhance antitumor immunity [211]. Beyond specific prebiotic supplements, broader dietary modifications, such as adopting a high-fiber diet, have been shown to create a gut environment that is more conducive to the establishment and sustained activity of probiotic communities, thereby amplifying their immunomodulatory impact [212].

#### Optimized manufacturing and rigorous quality control

Ensuring that probiotics remain viable throughout their shelf life is also critical. Advances in production technologies, such as freeze-drying and spray drying, are employed to enhance the long-term stability of bacterial formulations [213–215]. Equally important is the establishment of stringent quality standards and cold-chain logistics to prevent significant viability losses during storage and transportation. The implementation of standardized, quality-controlled production processes is essential to guarantee that the probiotic products administered to patients contain a therapeutically effective and reliable number of live bacteria, thereby closing the gap between laboratory promise and clinical reality [216].

In summary, probiotics demonstrate significant potential in tumor immunomodulation through multi-layered mechanisms, ranging from maintaining intestinal ecological balance to directly regulating the cancer-immunity cycle. Although encouraging clinical results have been achieved when combined with immunotherapy, their dual-edged nature necessitates more precise and innovative application strategies. Future research should focus on elucidating strain-specific mechanisms, developing next-generation engineered probiotics, and advancing personalized clinical applications to ultimately integrate probiotics safely and effectively into the arsenal of cancer immunotherapies.

### Engineered bacteria: precision-tailored living biologics

Among microbiota-targeted cancer immunotherapies, engineered bacteria have emerged as a distinct and promising therapeutic modality [39, 217, 218]. By exploiting the unique microenvironmental features of solid tumors, such as hypoxia and acidic pH, these microorganisms, particularly anaerobic and facultative anaerobic species, enable site-specific therapeutic interventions [219, 220]. Unlike conventional drugs, engineered bacteria function as autonomous drug factories, sustaining local delivery of immunomodulators such as cytokines and tumor antigens directly within the TME [219, 221]. Furthermore, synthetic biology enables the design of sophisticated genetic circuits that activate therapeutic payloads specifically in response to tumor-associated signals, minimizing off-target effects while maximizing local efficacy [222]. When combined with the intrinsic immunostimulatory properties of bacterial components like LPS, which activate PRRs to break immune tolerance, engineered bacteria emerge as a unique class of living therapeutics that synergize biological targeting with programmable precision for enhanced antitumor immunity [223, 224].

The development of therapeutic bacteria relies on two fundamental pillars: chassis selection and genetic circuit design. The choice of bacterial chassis involves a critical balance between tumor-killing capability and safety profile. Commonly used chassis include attenuated strains of Salmonella typhimurium [225], engineered Escherichia coli Nissle 1917 [226], safety-modified Listeria monocytogenes [227], and selected species of Bifidobacterium [228] and Clostridium [229], each providing distinct benefits in tumor targeting, immune modulation, and therapeutic payload delivery [230]. These chassis can be further optimized through various strategies. One approach involves introducing nutrient auxotrophies that restrict bacterial proliferation to TME regions rich in specific metabolites. For example, engineering leucine and arginine auxotrophies enables selective bacterial propagation in tumor regions abundant with these amino acids [231]. Similarly, constructing purine-auxotrophic Salmonella typhimurium VNP20009 allows the bacteria to thrive specifically in the purine-rich tumor niche [232]. Another strategy employs surface display of synthetic adhesion proteins to enhance tumor targeting. This can be achieved by engineering bacteria to express arginine-glycine-aspartic acid-containing peptides that specifically recognize αvβ3 integrin overexpressed on tumor cells [233]. Alternatively, engineered EcN coexpressing anti-PD-L1 and anti-CD9 nanobodies, enables spatiotemporal release of nanobodies targeting tumor-derived exosomes and improving antitumor immunity [234].

The engineering sophistication is primarily achieved through advanced genetic circuits that enable precise spatiotemporal control over therapeutic payload delivery. These systems encompass three main categories: (i) exogenous signal-triggered circuits that respond to external stimuli, including chemical inducers like tetracycline [235], optogenetic systems such as near-infrared light [236], and temperature-sensitive promoters activated by focused ultrasound [237]; (ii) autonomous bacterial signal-responsive circuits that utilize intrinsic mechanisms, exemplified by quorum sensing systems where LuxI/LuxR regulatory networks control synchronized lysis circuits for timed drug release [238], and intracellular niche-responsive circuits that activate only upon host cell invasion [239]; (iii) TME-responsive circuits that exploit pathological conditions, including hypoxia-sensing systems [240], acidity-responsive circuits [241] and lactate-sensing circuits based on LldR regulators [242]. The strategic implementation of these genetic control systems, particularly through combinatorial approaches employing logic gates, allows for unprecedented precision in restricting therapeutic activity to the tumor site while minimizing off-target effects.

Leveraging the aforementioned design strategies, engineered bacteria have been applied through several innovative paradigms to enhance anti-tumor immunity. First, they can locally produce immunomodulatory cytokines and chemokines within the TME. For example, Salmonella strains engineered to express the chemokine CCL21 remodel the immunosuppressive landscape by recruiting naive T cells and DCs, ultimately facilitating the formation of tertiary lymphoid structures within tumors [243]. Second, engineered bacteria can achieve localized delivery of ICIs, maximizing therapeutic efficacy while minimizing systemic toxicity. Engineered Escherichia coli Nissle secretes single-domain nanobodies targeting PD-L1 and CTLA-4 [244]. These nanobodies exhibit superior tumor penetration and maintain high intratumoral concentrations through continuous bacterial production, thereby activating T cells and promoting tumor regression in preclinical models without the irAEs typically associated with systemic ICIs administration. Third, the in-situ vaccination approach employs engineered bacteria to deliver tumor antigens directly to antigen-presenting cells within the TME. Listeria monocytogenes expressing antigens such as mesothelin efficiently engages both MHC-I and MHC-II presentation pathways, stimulating robust CD4⁺ and CD8⁺ T cell responses and has demonstrated promising results in clinical trials for pancreatic cancer [245]. Complementarily, a Synchronized Lysis Circuit implemented in E. coli enables controlled release of CD47-blocking nanobodies [246]. This disrupts the CD47-mediated anti-phagocytic signal and promotes macrophage-mediated phagocytosis of tumor cells by enhancing the recognition of malignant cells. Finally, combination strategies utilizing prodrug conversion provide localized chemotherapy delivery. For instance, Salmonella expressing cytosine deaminase converts systemically administered 5-fluorocytosine into the active chemotherapeutic 5-fluorouracil within tumors [247]. This approach achieves targeted tumor cell killing and induces immunogenic cell death, which releases tumor antigens and danger signals that synergize with immunotherapy to stimulate comprehensive anti-tumor immune responses. Collectively, these diverse paradigms highlight the versatility of engineered bacteria in overcoming the immunosuppressive TME through localized, controlled immune modulation, positioning them as promising platforms for the next generation of cancer immunotherapies.

Despite the immense promise, the clinical translation of engineered bacteria faces several fundamental challenges that must be systematically addressed. First, biosafety at the patient level remains the primary concern, particularly the risk of systemic infection in immunocompromised patients. Although attenuation strategies provide an initial safety layer, more sophisticated biocontainment systems have been developed to further restrict bacterial survival and activity within the host [248]. These include multiple auxotrophies that limit bacterial proliferation in healthy tissues [231], genetically encoded kill switches enabling controlled self-destruction [249], and genetic firewall designs that render bacterial viability dependent on synthetic amino acids absent from natural environments [250]. Second, manufacturing and quality control present unique challenges for living therapeutics. Unlike conventional drugs, maintaining the viability, genetic stability, and purity of engineered bacterial batches at scale requires sophisticated processes that can prevent the emergence of revertant strains while ensuring consistent therapeutic potency [248]. Third, host immune clearance poses a significant delivery barrier, as pre-existing antibodies or rapidly mounted adaptive immune responses can eliminate bacterial vectors before they establish effective tumor colonization, necessitating careful balancing of immunosuppressive strategies with infection risks [240, 251]. Fourth, tumor heterogeneity leads to variable colonization efficiency across different tumor types and even within different regions of the same tumor [252, 253]. Physical barriers like fibrosis and necrosis, along with variable immune infiltration, create inconsistent microenvironments for bacterial settlement, ultimately affecting therapeutic delivery uniformity and patient response predictability. Finally, regulatory frameworks for these innovative therapeutics remain in development, as engineered bacteria occupy an uncertain space between live biotherapeutic products, gene therapies, and conventional drugs [254]. Regulatory agencies like the FDA and EMA are establishing new evaluation criteria for assessing the safety, efficacy, and environmental impact of these self-replicating entities.

Beyond patient-specific safety considerations, engineered bacteria also introduce broader population-level and environmental biosafety concerns [254, 255]. Unintended dissemination to healthy individuals or natural ecosystems through bodily excretion or environmental exposure remains a key focus of regulatory scrutiny. Importantly, contemporary biocontainment strategies are not intended to enforce absolute confinement, but rather to establish clearly defined biological limits on survival, transmission, and evolution outside the therapeutic context [236, 252, 256]. Once outside their target environment, engineered bacteria are designed to rapidly lose viability, functional gene expression, or replicative capacity, thereby preventing sustained spread and horizontal gene transfer. From a regulatory standpoint, this design philosophy shifts the focus from eliminating exposure risk to ensuring predictable, self-limiting behavior following dissemination, providing a practical basis for balancing clinical innovation with public and environmental safety.

The clinical advancement of engineered bacteria will be propelled by several key research directions that address existing limitations while unlocking novel therapeutic capabilities. First, strategic integration with established treatment modalities represents the most immediate path to clinical impact. Engineered bacteria can be optimally combined with ICIs to convert immunologically “cold” tumors into T-cell-inflamed environments [257, 258], while simultaneous exploration with chemotherapy, radiotherapy, and oncolytic viruses may yield powerful synergistic antitumor effects [259]. Second, the development of advanced biomaterial-bacteria hybrids opens new therapeutic dimensions [241, 260, 261]. For example, a recently reported NIR-II responsive hybrid system integrates genetically engineered bacteria with nanomaterials to achieve photothermal ablation and enhanced immunotherapy upon NIR irradiation [262]. Third, the evolution toward increasingly sophisticated genetic circuits will enable unprecedented precision [240, 242, 263]. Next-generation systems incorporating Boolean logic gates will ensure therapeutic activation only under multiple tumor-specific conditions [264, 265], while advances in personalized medicine will facilitate the creation of customized bacterial therapies tailored to individual patients’ tumor molecular signatures. Finally, the exploitation of bacterial-derived vesicles as non-replicating delivery vehicles offers a promising alternative strategy [266–268]. Engineered outer membrane vesicles and other nanoparticles are being developed to display tumor-targeting ligands and deliver immunostimulatory cargoes while circumventing the safety concerns associated with live bacterial administration.

In conclusion, engineered bacteria represent a paradigm shift in cancer treatment, transforming a historical observation that bacteria can infect tumors into a cutting-edge precision medicine platform. By harnessing the tools of synthetic biology, we are learning to program these simple organisms to become intelligent allies in the fight against cancer. They are no longer just passive supplements but active, sensing, and responding therapeutic agents. Despite significant challenges in safety, manufacturing, and regulation, the rapid innovation in this field suggests that engineered living medicines are poised to become integral components of the next generation of cancer immunotherapies, ultimately offering new hope for patients with currently untreatable malignancies.

## Challenges and future perspectives

The burgeoning field of oncomicrobiomics stands at a critical juncture, poised between compelling scientific evidence and meaningful clinical translation. While the potential of microbiota-targeted interventions to enhance cancer immunotherapy is undeniable, their path to standardized clinical implementation faces conceptual, technical, and practical challenges.

### From correlation to causation: defining mechanistic precision

The fundamental challenge remains establishing definitive causal relationships between specific microbial features and immunotherapy outcomes [269]. This requires sophisticated experimental approaches including gnotobiotic mouse models colonized with defined microbial communities, in vitro immune cell-microbe coculture systems, and detailed mechanistic studies of microbial metabolites and their cellular targets. The field must move beyond simply identifying “good” and “bad” bacteria toward understanding the precise molecular mechanisms through which microbes influence host immunity [270]. This includes characterizing the specific bacterial strains, their active components, the host receptors they engage, and the downstream signaling pathways they activate. Only with this level of mechanistic clarity can truly rational, targeted microbiota-based interventions be developed, rather than relying on broad, ecosystem-level interventions.

### Microbial biomarkers: from composition to function

The development of reliable predictive biomarkers represents another critical challenge. Current studies focus primarily on taxonomic composition, but this approach is limited by the high interpersonal variability of microbial communities and the functional redundancy across different bacterial taxa [271, 272]. Future biomarker development must therefore prioritize functional capacity over taxonomic identity, employing metagenomic, metatranscriptomic, and metabolomic analyses to identify conserved functional pathways associated with treatment response. Ideally, biomarkers should predict not only the likelihood of therapeutic response but also the risk of irAEs, thereby enabling truly personalized treatment decisions [273]. Standardization of sampling time points is also essential. Although baseline samples hold promise for prediction, serial sampling during treatment may provide dynamic insights into ecological shifts that correlate with evolving immune responses. Ultimately, validated microbial biomarkers must be integrated with established host and tumor determinants to create comprehensive multidimensional predictive models that can effectively guide clinical decision-making.

### The personalization paradox: universal solutions versus individualized approaches

A fundamental challenge exists between developing universal microbial therapeutics and the inherently individualized nature of human microbial ecosystems. The same bacterial strain can exert markedly different effects depending on the ecological context of the recipient’s gut environment, including dietary patterns [274] and genetic background [157]. Consequently, effective interventions may need to be personalized based on an individual’s baseline microbiota composition and functional capacity. However, such personalization introduces substantial challenges for therapeutic development, regulatory evaluation, and clinical implementation. Potential strategies to reconcile this paradox include designing defined microbial consortia that retain activity across diverse ecological contexts, identifying patient stratification markers that predict responsiveness to specific interventions, and developing modular platforms that can be adapted to individual microbial deficiencies. A particularly promising approach is to stratify patients according to the functional potential of their microbiota rather than its taxonomic composition, thereby enabling more tailored and mechanistically grounded intervention strategies [275, 276].

### Technical and standardization hurdles

The technical challenges in microbiome research and the development of microbiome-based interventions are substantial and multifaceted. During sample processing, variations in collection methods, storage conditions, DNA extraction protocols, and bioinformatic pipelines can markedly impact results and hinder comparability across studies [275, 277]. Consequently, consensus guidelines for standardized methodologies are urgently needed. For live biotherapeutic products, manufacturing poses unique challenges in maintaining the viability, stability, and consistency of complex biological materials [276]. Advances in formulation science are required to ensure optimal delivery of microbes to the appropriate intestinal regions while protecting them from gastric acid and bile acids exposure. Dosage determination differs substantially from that of conventional pharmaceuticals, as microbes can proliferate and interact dynamically with the existing microbial ecosystem [278]. Developing pharmacodynamic metrics that accurately reflect microbial engraftment and functional effects represents an innovative area of research that will be critical for successful clinical translation [276].

### Clinical trial design innovation

Traditional clinical trial designs are often poorly suited for evaluating microbiome-based interventions, as their effects may be modified by the baseline composition of the host microbiota and may require endpoints distinct from those used in conventional therapies [279, 280]. To address these challenges, innovative trial designs such as adaptive platforms, enrichment strategies based on microbial biomarkers, and crossover approaches have been proposed to accelerate development [279, 281]. Combination strategies introduce additional complexity, as microbiota-directed interventions may interact synergistically or antagonistically with various immunotherapies and conventional treatments [282]. Moreover, the optimal timing and sequencing of microbial interventions relative to immunotherapy cycles remain undefined [283]. Large-scale, collaborative efforts incorporating standardized outcome measures and harmonized sample collection protocols will be essential to generate robust, reproducible evidence across diverse patient populations.

### Safety and regulatory considerations

As living therapeutics, microbiome-based interventions pose unique safety challenges, including the potential for horizontal gene transfer [284], long-term ecological alterations [285], and unintended immune activation [286]. While FMT has demonstrated relative safety in treating Clostridium difficile infection, its safety profile in immunocompromised cancer patients requires ongoing vigilance. Regulatory pathways for microbiome-based therapies remain under active development, with agencies working to establish appropriate frameworks for evaluating these complex biological products [287]. Standards for adequate characterization, potency assays, and quality control of microbial consortia are still evolving [279]. In addition, ethical considerations such as donor screening, informed consent for novel biological therapies, and equitable access to emerging treatments, require sustained attention as the field progresses [275].

## Conclusion

The recognition that gut microbiota profoundly influence responses to cancer immunotherapy represents a paradigm shift in oncology. From initial correlative observations, the field has rapidly advanced to demonstrate causal relationships and develop innovative intervention strategies. Each approach offers distinct advantages and challenges, and their ultimate clinical utility will depend on patient-specific factors including baseline microbiota composition and disease characteristics. Looking ahead, integrating microbiome-targeted approaches into cancer immunotherapy holds tremendous promise for broadening the benefit of these treatments to a larger patient population.

## Data Availability

No datasets were generated during the current study.

## Abbreviations

AhR: Aryl hydrocarbon receptor
AR: Androgen receptor
ASC: Apoptosis-associated speck-like protein containing a CARD
AEs: Adverse events
BAs: Bile acids
CBM588: Clostridium butyricum MIYAIRI 588
CA: Cholic acid
cAMP: Cyclic adenosine monophosphate
CAR: Chimeric antigen receptor
CBM: Clostridium butyricum
CCL: C-C motif chemokine ligand
CD: Cluster of differentiation
CDCA: Chenodeoxycholic acid
CTLA-4: Cytotoxic T-lymphocyte-associated protein 4
DCA: Deoxycholic acid
FMT: Fecal microbiota transplantation
Foxp3: Forkhead box P3
FXR: Farnesoid X receptor
Gly: Glycine
HDAC: Histone deacetylase
ICB: Immune checkpoint blockade
ICIs: Immune checkpoint inhibitors
IDO1: Indoleamine 2,3-dioxygenase 1
IFN-γ: Interferon-γ
IKK: IκB kinase
IL: Interleukin
irAEs: Immune-related adverse events
Kyn: Kynurenine
LCA: Lithocholic acid
LPS: Lipopolysaccharide
MET4: Microbial ecosystem therapeutic 4
NCOR: Nuclear receptor corepressor
NF-κB: Nuclear factor kappa B
NIR: Near-infrared
NLRP3: NOD-, LRR- and pyrin domain-containing protein 3
NSCLC: Non-small cell lung cancer
OS: Overall survival
PCD: Programmed cell death
PD-1: Programmed cell death protein 1
PD-L1: Programmed cell death ligand 1
PFS: Progression-free survival
PKA: Protein kinase A
PPI: Proton pump inhibitor
PRRs: Pattern recognition receptors
QC: Quality control
RORγt: Retinoic acid-related orphan receptor gamma t
SCFAs: Short-chain fatty acids
STAT1: Signal transducer and activator of transcription 1
TAMs: Tumor-associated macrophages
TCR: T cell receptor
TGF-β: Transforming growth factor-β
TILs: Tumor-infiltrating lymphocytes
TME: Tumor microenvironment
TNF-α: Tumor necrosis factor-α
Treg: Regulatory T cell
TLS: Tertiary lymphoid structures
Trp: Tryptophan
VDR: Vitamin D receptor
